# Highly Robust Adaptive Sliding Mode Trajectory Tracking Control of Autonomous Vehicles

**DOI:** 10.3390/s23073454

**Published:** 2023-03-25

**Authors:** Fengxi Xie, Guozhen Liang, Ying-Ren Chien

**Affiliations:** 1 Department of Electrical Engineering and Computer Science, Technische Universität Berlin, 10623 Berlin, Germany; 2 Department of Electrical Engineering, National Ilan University, Yilan 260007, Taiwan

**Keywords:** autonomous vehicles, trajectory tracking, high robustness, vector field guidance law, sliding mode control, improved particle swarm optimization, improved grey wolf optimization

## Abstract

Autonomous driving technology has not yet been widely adopted, in part due to the challenge of achieving high-accuracy trajectory tracking in complex and hazardous driving scenarios. To this end, we proposed an adaptive sliding mode controller optimized by an improved particle swarm optimization (PSO) algorithm. Based on the improved PSO, we also proposed an enhanced grey wolf optimization (GWO) algorithm to optimize the controller. Taking the expected trajectory and vehicle speed as inputs, the proposed control scheme calculates the tracking error based on an expanded vector field guidance law and obtains the control values, including the vehicle’s orientation angle and velocity on the basis of sliding mode control (SMC). To improve PSO, we proposed a three-stage update function for the inertial weight and a dynamic update law for the learning rates to avoid the local optimum dilemma. For the improvement in GWO, we were inspired by PSO and added speed and memory mechanisms to the GWO algorithm. Using the improved optimization algorithm, the control performance was successfully optimized. Moreover, Lyapunov’s approach is adopted to prove the stability of the proposed control schemes. Finally, the simulation shows that the proposed control scheme is able to provide more precise response, faster convergence, and better robustness in comparison with the other widely used controllers.

## 1. Introduction

In recent years, researchers have demonstrated that autonomous vehicles (AVs) can possibly reduce road accident rates and enhance transportation efficiency [1,2,3], thus drawing massive attention from both industrial and academic fields.

Trajectory tracking is one of the basic functions of AVs, which aims to follow a desired trajectory within a certain period of time and then to maintain motion stability continuously [4]. Control theory is a fundamental element in various domains [5]. Recently, different kinds of methods and controllers associated with trajectory tracking have been developed. For PID, Han et al. proposed a lateral path-following controller, which can fine-tune the control parameters by integrating a neural network [6]. In regards to multi-task control (e.g., tracking precision, driving comfort, and ride stability), due to the limited capacity for handling several control objectives, a PID controller is not the ideal control scheme for the trajectory tracking of AVs [7,8,9,10,11]. For fuzzy logic control, El et al. designed a Takagi–Sugeno fuzzy controller, in which the stability was proved by integrating linear matrix inequalities in a Lyapunov stability analysis [12]. In addition to PID control, SMC is also widely used in improving system robustness. Compared with PID, SMC requires fewer parameters to be adjusted and has a faster response. It is considered a powerful control technique for suppressing external disturbances [13,14,15,16,17]. He et al. proposed a backstepping SMC controller to collision-free path tracking [18]. Guo et al. introduced an adaptive fuzzy sliding-mode controller for the steering and brake control [19]. In order to achieve trajectory tracking of an unmanned agricultural tractor, Matveev et al. developed a nonlinear SMC control strategy, which showed a robust performance [20]. However, SMC has a significant drawback, namely chattering, and a number of methods have been proposed to effectively mitigate the chattering effect and to improve the robustness of the system [21,22,23,24,25].

For trajectory modeling, input parameters include vehicle states such as velocity, orientation angle, and acceleration. However, these states could only be obtained through specific equipment. To this end, researchers have designed robust estimators by integrating cameras, global navigation satellite system (GNSS), and inertial measurement unit (IMU) [26,27,28,29,30]. These techniques could be used in the future whole-vehicle test verification of our proposed approach.

For all of the aforementioned control schemes, to achieve good performance, the gain of their controller needs to be manually adjusted, and in different application scenarios, the gain value of the controller can be very different and people may spend a lot of time on fine-tuning the parameters. In order to overcome this difficulty and to make the control scheme more effective, an optimization algorithm can be carefully designed for adaptive control parameter tuning. Traditional optimization techniques, such as gradient-based methods, may not always work efficiently or may even fail to converge to a global optimum. In contrast, PSO and GWO are population-based optimization algorithms that mimic the behavior of a swarm or a pack of animals, respectively, aiming to search for the global optimum in a multidimensional solution domain. Firstly, designing a good cost function is an important part of the optimization algorithm [31]. Fateh et al. applied PSO to optimize robust control of robot manipulators [32]. A fuzzy controller can also be tuned by PSO [33,34]. To obtain a robust and adaptive PID controller, Elkaranshway et al. adopted PSO for parameter fine-tuning [35]. However, the inertial weight and learning rates of the PSO algorithm introduced in previous work were usually set to a constant, often leading to a local optimum dilemma. While PSO is commonly used in optimizing control schemes, there are also other algorithms that can be adopted, such as the GWO. As a meta-heuristic optimization algorithm, GWO has been shown to have better exploration–exploitation trade-off, faster convergence, and the ability to handle multiple objectives when compared with PSO [36]. GWO is also less susceptible to premature convergence than PSO [37]. Furthermore, GWO has been improved with the use of crossover operators and hybridization with other algorithms, such as the sine cosine algorithm, leading to even better performance. Therefore, GWO may be an alternative for optimizing control schemes.

In this paper, we combine the vector field guidance law approach with trajectory tracking control. In recent years, the vector field approach has been well introduced to provide a better solution for many control problems [38]. By creating a vector field around the target path, we ensure that the tracking error approaches zero asymptotically, even if the vehicle undergoes external disturbances [39]. More recently, scholars have been trying to extend the vector field approach in different path-control-related problems [40,41,42].

However, it is noteworthy that the fundamental mission of autonomous vehicles is to ensure passengers’ safety [43]. Therefore, the controller should be adaptable. Even in dangerous scenarios (driving at high speed on complex trajectories), the controller can autonomously tune its gain and minimize errors. Current studies mainly focus on path following and do not meet the adaptive requirements of autonomous vehicles. Additionally, the predefined curves in previous studies are simple and differ from practical traffic scenarios.

Given this problem, this paper implements a high-accuracy and adaptive trajectory tracking controller by integrating sliding mode control and novel evolutionary optimization algorithms. This study has three contributions: (1) A vector field guidance law has been designed and extended from a simple curve to a sine trajectory and a polynomial trajectory, which assembles the lane-change trajectory in practical traffic scenarios. (2) A sliding mode controller of velocity and angle are designed separately to ensure better performance, and improved optimization algorithms, named IPSO and PGWO, are designed. (3) We integrate the designed vector field guidance law, optimization algorithm, and sliding mode controllers to create an adaptive control scheme and formulate the corresponding Lyapunov function to demonstrate system stability. We compare the proposed control scheme against other commonly employed methods. The simulation results prove that our design outperforms traditional methods in complex scenarios and considerably enhances the system’s robustness.

Regarding the arrangement of this paper, Section 2 introduces the vehicle kinematic model. In Section 3, the structure and the theory of optimized control strategy are described. Section 4 presents and analyzes the simulation results of different controllers. Finally, Section 5 summarizes the workflow of this study and discusses possible future improvements.

## 2. Preliminaries

**Figure 1:**
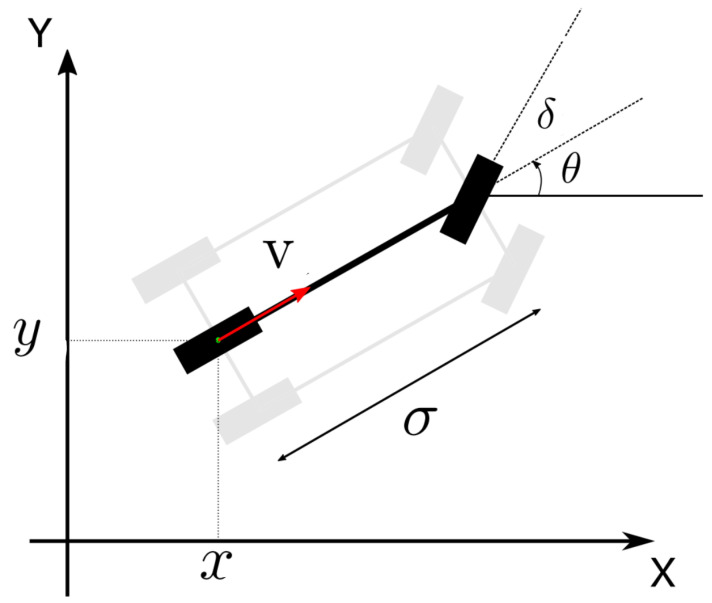
Vehicle model.

Referring to Figure 1, we obtain the vehicle’s kinematics model:

(1) $$\begin{array}{cc} \dot{x} & =v\cos\left(\theta \right)\\ \dot{y} & =v\sin\left(\theta \right)\\ \dot{\theta } & =\sigma^{-1}v\tan\left(\delta \right)\\ \dot{v} & =F \end{array}$$

where σ>0 represents the distance between the front axle and the rear axle of this vehicle. To better describe the trajectory of an autonomous vehicle, we use the midpoint of the rear axle as the reference point, which can also be represented as $\left(x,y\right)$. In an inertial frame with Cartesian coordinates (X,Y), *x* and *y* are the longitudinal position and lateral position of the vehicle, respectively. *v* is the vehicle velocity at the point $\left(x,y\right)$, while $\theta \in \left(-\frac{\pi }{2},\frac{\pi }{2}\right)$ is the angular orientation of the vehicle with respect to the *X* axis. In fact, lateral control of the vehicle is achieved by changing the steering angle of the car. Here, we derive the relationship between the orientation angle θ and the steering angle of the front wheels by analyzing the kinematics of the vehicle. *F* represents the acceleration of the vehicle.

## 3. Controller Design

### 3.1. Control Scheme of Proposed Controller

**Figure 2:**
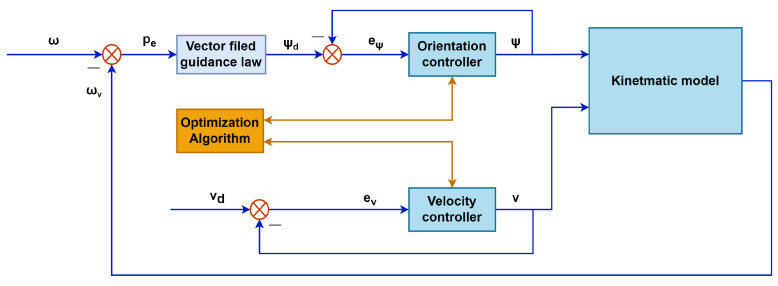
Control scheme of the proposed controller.

The architecture of the IPSO-SM controller is shown in Figure 2. The desired trajectory and velocity are used as input in the model. With the help of the expanded vector field guidance method, a set of desired yaw angles will be generated from the input trajectory. The orientation and velocity controller will output the optimal yaw angle and velocity according to the current angle error and velocity error, respectively, under the optimization of IPSO. Then, after the calculation of the vehicle kinematic model, the current position of the vehicle will be the output.

### 3.2. Vector Field Guidance Law

The vector field guidance law has been expanded from the simple curve [39] to the complex curve in this paper. For a certain trajectory, Ꮗ, ${Ꮗ}_{c}$ is defined as the closest point and ${Ꮗ}_{v}$ is the location of the vehicle; then, the distance vector $\nu_{1}\left({Ꮗ}_{c}\right)$ and the scalar distance $\nu_{1}\left({Ꮗ}_{c}\right)$ are defined as

(2) $$\begin{array}{cc} \nu_{1}\left({Ꮗ}_{c}\right) & ={Ꮗ}_{c}-{Ꮗ}_{v}\\ \nu_{1}\left({Ꮗ}_{c}\right) & =\left|\right|{Ꮗ}_{c}-{Ꮗ}_{v}\left|\right| \end{array}$$

with $\nu_{2}\left({Ꮗ}_{c}\right)$ as the curve’s tangent vector at ${Ꮗ}_{c}$.

To facilitate the calculation, two operators are defined here.

(3) $$\begin{array}{cc} \kappa_{1} & =\left(2/\pi \right)\arctan\left(k_{f}\nu_{1}\right)\\ \kappa_{2} & =\sqrt{1-\kappa_{1}^{2}} \end{array}$$

where $k_{f}\in R^{+}$.

The guidance vector ${Ꮗ}_{v}$ is designed as

(4) $$\begin{array}{c} \Theta \left({Ꮗ}_{c}\right)=-\kappa_{1}\frac{\nu_{1}\left({Ꮗ}_{c}\right)}{\nu_{1}\left({Ꮗ}_{c}\right)}+\kappa_{2}\nu_{2}\left({Ꮗ}_{c}\right) \end{array}$$

To achieve accurate angle control, the desired yaw angle is calculated for each position of the vehicle in motion. Since Θ is a series of vectors forming a trajectory, Θ(1) and Θ(2) are used to denote the first and second values of trajectory vector, respectively. Then, the desired yaw angle is

(5) $$\psi_{d}=\arctan\left(\frac{\Theta (1)}{\Theta (2)}\right)$$

where the subscript d means a desired value, which should be tracked. The yaw angle of the vehicle is assumed to be capable of tracking the desired yaw angle $\psi_{d}$. Substituting (4) into (5) yields the derivative of position error $p_{e}$

(6) $$\begin{array}{cc} {\dot{p}}_{e} & =V\sin\left(\psi_{d}-\psi_{p}\right)\\ \; & =V\sin\left(\psi_{d}-\arctan\left(\frac{\nu_{2}\left(1\right)}{\nu_{2}\left(2\right)}\right)\right). \end{array}$$

where $\psi_{p}$ denotes the current yaw angle. Then, to simplify the calculation, we define

(7) $$\nu_{3}=\kappa_{1}\frac{\nu_{1}\left({Ꮗ}_{c}\right)}{\nu_{1}\left({Ꮗ}_{c}\right)}+\left(1-\kappa_{2}\right)\nu_{2}\left({Ꮗ}_{c}\right)$$

Therefore, (6) can be simplified to (8):

(8) $${\dot{p}}_{e}=-V\sin\left(\arctan\left(\frac{\nu_{3}\left(1\right)}{\nu_{3}\left(2\right)}\right)\right)$$

To prove the stability of the method, we use the position error $p_{e}$ to design a Lyapunov candidate $V_{1}$:

(9) $$V_{1}=\frac{1}{2}p_{e}^{2},$$

where $p_{e}^{2}$ is always > 0 when $p_{e}$ is not equal to zero. Additionally, according to (8), because $\nu_{1}\left({Ꮗ}_{c}\right)$ and $\nu_{2}\left({Ꮗ}_{c}\right)$ are a set of orthogonal vectors, it is then $\arctan\left(\frac{\nu_{3}\left(1\right)}{\nu_{3}\left(2\right)}\right)<\pi /2$; then, $\sin\left(\psi_{d}-\arctan\left(\frac{\nu_{2}\left(1\right)}{\nu_{2}\left(2\right)}\right)\right)>0$. Thus, ${\dot{p}}_{e}<0$ holds. Therefore, $\exists \sigma_{1}>0$, the derivative of $V_{1}$ can be obtained as

(10) $$\begin{array}{cc} {\dot{V}}_{1} & =p_{e}{\dot{p}}_{e}\\ \; & \leq -\sigma_{1}V_{1}. \end{array}$$

### 3.3. Orientation Angle Controller Design

To achieve lateral control of the vehicle, it is necessary to minimize the yaw error. Define ψ as the current yaw angle of the vehicle, so the angle error $e_{\psi ,1}$ is

(11) $$e_{\psi ,1}=\psi -\psi_{d}$$

Similar to Section 3.2, a Lyapunov candidate is considered as (12):

(12) $$V_{2}=\frac{1}{2}e_{\psi ,1}^{2}$$

The differential of this candidate is described as (13):

(13) $$\begin{array}{cc} {\dot{V}}_{2} & =e_{\psi ,1}{\dot{e}}_{\psi ,1}\\ \; & =e_{\psi ,1}\left(\dot{\psi }-{\dot{\psi }}_{d}\right) \end{array}$$

In order to bring the defined error closer to the true error, here, we introduce the nominal error. Define the nominal yaw angle error as (14), where $k_{\psi ,1}>0$.

(14) $$e_{\psi ,2}=\dot{\psi }-{\dot{\psi }}_{d}+k_{\psi ,1}e_{\psi ,1}$$

Combining Equations (13) and (14) yields

(15) $${\dot{V}}_{2}=e_{\psi ,1}e_{\psi ,2}-k_{\psi ,1}e_{\psi ,1}^{2}$$

Based on the principle of sliding mode control, we define the sliding mode surface $s_{1}$, which consists of angle errors

(16) $$s_{1}=k_{\psi ,2}e_{\psi ,1}+e_{\psi ,2}$$

where $k_{\psi ,2}$ is the sliding mode surface parameter and $k_{\psi ,2}>0$.

According (11) and (14), the differential of $e_{\psi ,1}$ can be described as (17):

(17) $$\begin{array}{cc} {\dot{e}}_{\psi ,1} & =\dot{\psi }-{\dot{\psi }}_{d}\\ \; & =e_{\psi ,2}-k_{\psi ,1}e_{\psi ,1} \end{array}$$

To prove the stability of the orientation angle controller, a Lyapunov candidate $V_{3}$ for this controller should be designed in order to ensure that the Lyapunov function decreases along the sliding mode surface and to reduce the number of variables, and simplifying the expression of the Lyapunov function, $V_{3}$ should include $s_{1}$ and $V_{2}$. Therefore, it is considered as follows:

(18) $$V_{3}=V_{2}+\frac{1}{2}s_{1}^{2}$$

After differentiating, we substitute (16) and (17) into this and then derive (19):

(19) $$\begin{array}{cc} {\dot{V}}_{3} & ={\dot{V}}_{2}+\frac{1}{2}{\dot{s}}_{1}^{2}\\ \; & =e_{\psi ,1}e_{\psi ,2}-k_{\psi ,1}e_{\psi ,1}^{2}+s_{1}{\dot{s}}_{1}\\ \; & =e_{\psi ,1}e_{\psi ,2}-k_{\psi ,1}e_{\psi ,1}^{2}+s_{1}\left(k_{\psi ,2}{\dot{e}}_{\psi ,1}+{\dot{e}}_{\psi ,2}\right)\\ \; & =e_{\psi ,1}e_{\psi ,2}-k_{\psi ,1}e_{\psi ,1}^{2}+s_{1}\left[k_{\psi ,2}\left(e_{\psi ,2}-k_{\psi ,1}e_{\psi ,1}\right)+\ddot{\psi }-{\ddot{\psi }}_{d}+k_{\psi ,1}{\dot{e}}_{\psi ,1}\right] \end{array}$$

In order to speed up the convergence of the sliding surface and to make the whole process smoother and less chattering, the reaching law is designed as

(20) $${\dot{s}}_{1}=-k_{\psi ,3}{\left|s_{1}\right|}^{\alpha_{1}}\mathrm{sgn}\left(s_{1}\right)-\left(\frac{1}{2k_{\psi ,2}}+e^{f_{\psi }\left(s_{1}\right)}{\left|s_{1}\right|}^{\beta_{1}}\right)s_{1},$$

in which $k_{\psi ,3}>0$, $1>\alpha_{1}>0$, $1>\beta_{1}>0$, and $f_{\psi }\left(s_{1}\right)$ is designed as

(21) $$f_{\psi }\left(s_{1}\right)=\left\{\begin{array}{c} |s_{1}\left|(\right|s_{1}|-\Delta_{\psi }),|s_{1}|\geq \Delta_{\psi }\\ -\frac{1-|s_{1}|/\Delta_{\psi }}{|s_{1}|+\Delta_{\psi }},\left|s_{1}\right|<\Delta_{\psi } \end{array}\right.,$$

where $\Delta_{\psi }$ denotes the thickness of the sliding mode surface.

Combining the above equations, the sliding mode controller is designed as

(22) $$u_{d}=-k_{\psi ,3}{\left|s_{1}\right|}^{\alpha_{1}}\mathrm{sgn}\left(s_{1}\right)-\left(\frac{1}{2k_{\psi ,2}}+e^{f_{\psi }\left(s_{1}\right)}{\left|s_{1}\right|}^{\beta_{1}}\right)s_{1}-k_{\psi ,2}\left(e_{\psi ,2}-k_{\psi ,1}e_{\psi ,1}\right)+{\ddot{\psi }}_{d}-k_{\psi ,1}{\dot{e}}_{\psi ,1}$$

Then, the desired steering angle of the front wheels can be designed as

(23) $$\delta_{d}=\arctan\left(v^{-1}\sigma u_{d}\right)$$

Then, substituting (20) into (19), the Lyapunov candidate $V_{3}$ can be described as

(24) $$\begin{array}{cc} {\dot{V}}_{3} & =e_{\psi ,1}e_{\psi ,2}-k_{\psi ,1}e_{\psi ,1}^{2}+s_{1}\left[-k_{\psi ,3}{\left|s_{1}\right|}^{\alpha_{1}}\mathrm{sgn}\left(s_{1}\right)-\left(\frac{1}{2k_{\psi ,2}}+e^{f_{\psi }\left(s_{1}\right)}{\left|s_{1}\right|}^{\beta_{1}}\right)s_{1}\right]\\ \; & =e_{\psi ,1}e_{\psi ,2}-k_{\psi ,1}e_{\psi ,1}^{2}-k_{\psi ,3}|s_{1}{|}^{\alpha_{1}}|s_{1}|-\frac{1}{2k_{\psi ,2}}s_{1}^{2}-e^{f_{\psi }\left(s_{1}\right)}{\left|s_{1}\right|}^{\beta_{1}}s_{1}^{2}\\ \; & =e_{\psi ,1}e_{\psi ,2}-k_{\psi ,1}e_{\psi ,1}^{2}-\frac{1}{2k_{\psi ,2}}{\left(k_{\psi ,2}e_{\psi ,1}+e_{\psi ,2}\right)}^{2}-k_{\psi ,3}|s_{1}{|}^{\alpha_{1}+1}-e^{f_{\psi }\left(s_{1}\right)}{\left|s_{1}\right|}^{\beta_{1}}s_{1}^{2}\\ \; & =-k_{\psi ,1}e_{\psi ,1}^{2}-\frac{1}{2k_{\psi ,2}}\left(k_{\psi ,2}^{2}e_{\psi ,1}^{2}+e_{\psi ,2}^{2}\right)-k_{\psi ,3}|s_{1}{|}^{\alpha_{1}+1}-e^{f_{\psi }\left(s_{1}\right)}{\left|s_{1}\right|}^{\beta_{1}}s_{1}^{2} \end{array}$$

According to (12) and (19), $V_{2}\geq 0$ and $V_{3}\geq 0$. Additionally, $k_{\psi ,1}>0$, $k_{\psi ,2}>0$, $k_{\psi ,3}>0$, and $e^{f_{\psi }\left(s_{1}\right)}{\left|s_{1}\right|}^{\beta_{1}}>0$. Therefore, the conclusion that $\exists \sigma_{3}>0$ can be given such that ${\dot{V}}_{3}\leq -\sigma_{3}V_{3}$.

### 3.4. Velocity Controller Design

The controller of velocity is similar to the orientation angle controller; however, in the orientation angle controller, the desired yaw angle of the vehicle is calculated by the guidance law, while the desired velocity of the vehicle here is defined artificially. Define the velocity error $e_{v,1}$ as (25), where v is the current velocity and $v_{d}$ is the desired velocity.

(25) $$e_{v,1}=v-v_{d}$$

For the velocity error, similar to Section 3.3, consider a Lyapunov candidate:

(26) $$V_{4}=\frac{1}{2}e_{v,1}^{2}$$

Differentiate it as

(27) $$\begin{array}{cc} {\dot{V}}_{4} & =e_{v,1}{\dot{e}}_{v,1}\\ \; & =e_{v,1}\left(\dot{v}-{\dot{v}}_{d}\right) \end{array}$$

Similar to the orientation controller, in order to improve the robustness of the controller, here, the nominal velocity error is defined as

(28) $$e_{v,2}=\dot{v}-{\dot{v}}_{d}+k_{v,1}e_{v,1}$$

where $k_{v,1}>0$.

Combining (27) and (28) yields (29), here, the differential of $V_{4}$ can be described with the velocity error and nominal velocity error.

(29) $${\dot{V}}_{4}=e_{v,1}e_{v,2}-k_{v,1}e_{v,1}^{2}$$

Define the sliding mode surface $s_{2}$, which consists of $e_{v,1}$ and $e_{v,2}$:

(30) $$s_{2}=k_{v,2}e_{v,1}+e_{v,2}$$

where $k_{v,2}$ is the sliding mode surface parameter and $k_{v,2}>0$.

According (25) and (28), differentiate $e_{v_{1}}$ as (31):

(31) $$\begin{array}{cc} {\dot{e}}_{v_{1}} & =\dot{v}-{\dot{v}}_{d}\\ \; & =e_{v,2}-k_{v,1}e_{v,1} \end{array}$$

Consider a Lyapunov candidate $V_{5}$, which consists of $V_{4}$ and $s_{2}$:

(32) $$V_{5}=V_{4}+\frac{1}{2}s_{2}^{2}$$

Combining (29), (30), and (32) yields

(33) $$\begin{array}{cc} {\dot{V}}_{5} & ={\dot{V}}_{4}+\frac{1}{2}{\dot{s}}_{2}^{2}\\ \; & =e_{v,1}e_{v,2}-k_{v,1}e_{v,1}^{2}+s_{2}{\dot{s}}_{2}\\ \; & =e_{v,1}e_{v,2}-k_{v,1}e_{v,1}^{2}+s_{2}\left(k_{v,2}{\dot{e}}_{v,1}+{\dot{e}}_{v,2}\right)\\ \; & =e_{v,1}e_{v,2}-k_{v,1}e_{v,1}^{2}+s_{2}\left[k_{v,2}\left(e_{v,2}-k_{v,1}e_{v,1}\right)+\ddot{v}-{\ddot{v}}_{d}+k_{v,1}{\dot{e}}_{v,1}\right] \end{array}$$

In order to speed up the convergence of the sliding surface and to make the whole process smoother and less chattering, the reaching law ${\dot{s}}_{2}$ is designed as

(34) $${\dot{s}}_{2}=-k_{v,3}{\left|s_{2}\right|}^{\alpha_{2}}\mathrm{sgn}\left(s_{2}\right)-\left(\frac{1}{2k_{v,2}}+e^{f_{v}\left(s_{2}\right)}{\left|s_{2}\right|}^{\beta_{2}}\right)s_{2}$$

in which $k_{v,3}>0$, $1>\alpha_{2}>0$, $1>\beta_{2}>0$, and $f_{v}\left(s_{2}\right)$ is designed as

(35) $$f_{v}\left(s_{2}\right)=\left\{\begin{array}{c} |s_{2}\left|(\right|s_{2}|-\Delta_{v}),|s_{2}|\geq \Delta_{v}\\ -\frac{1-|s_{2}|/\Delta_{v}}{|s_{2}|+\Delta_{v}},\left|s_{2}\right|<\Delta_{v} \end{array}\right.$$

in which $\Delta_{v}$ denotes the thickness of the sliding mode surface.

The sliding mode controller is designed as

(36) $$F_{d}=-k_{v,3}{\left|s_{2}\right|}^{\alpha_{2}}\mathrm{sgn}\left(s_{2}\right)-\left(\frac{1}{2k_{v,2}}+e^{f_{v}\left(s_{2}\right)}{\left|s_{2}\right|}^{\beta_{2}}\right)s_{2}-k_{v,2}\left(e_{v,2}-k_{1}e_{v,1}\right)+{\ddot{v}}_{d}-k_{v,1}{\dot{e}}_{1}$$

Then,

(37) $$\begin{array}{cc} {\dot{V}}_{5} & =e_{v,1}e_{v,2}-k_{v,1}e_{v,1}^{2}+s_{2}\left[-k_{v,3}{\left|s_{2}\right|}^{\alpha_{2}}\mathrm{sgn}\left(s_{2}\right)-\left(\frac{1}{2k_{v,2}}+e^{f_{v}\left(s_{2}\right)}{\left|s_{2}\right|}^{\beta_{2}}\right)s_{2}\right]\\ \; & =e_{v,1}e_{v,2}-k_{v,1}e_{v,1}^{2}-k_{v,3}|s_{2}{|}^{\alpha_{2}}|s_{2}|-\frac{1}{2k_{v,2}}s_{2}^{2}-e^{f_{v}\left(s_{2}\right)}{\left|s_{2}\right|}^{\beta_{2}}s_{2}^{2}\\ \; & =e_{v,1}e_{v,2}-k_{v,1}e_{v,1}^{2}-\frac{1}{2k_{v,2}}{\left(k_{v,2}e_{v,1}+e_{v,2}\right)}^{2}-k_{v,3}|s_{2}{|}^{\alpha_{2}+1}-e^{f_{v}\left(s_{2}\right)}{\left|s_{2}\right|}^{\beta_{2}}s_{2}^{2}\\ \; & =-k_{v,1}e_{v,1}^{2}-\frac{1}{2k_{v,2}}\left(k_{v,2}^{2}e_{v,1}^{2}+e_{v,2}^{2}\right)-k_{v,3}|s_{2}{|}^{\alpha_{2}+1}-e^{f_{v}\left(s_{2}\right)}{\left|s_{2}\right|}^{\beta_{2}}s_{2}^{2} \end{array}$$

According to (26) and (32), $V_{4}\geq 0$, $V_{5}\geq 0$. Additionally, $k_{v,1}>0$, $k_{v,2}>0$, $k_{v,3}>0$, and $e^{f_{v}\left(s_{2}\right)}{\left|s_{2}\right|}^{\beta_{2}}>0$. Therefore, the conclusion can be given that $\exists \sigma_{5}>0$ such that ${\dot{V}}_{5}\leq -\sigma_{5}V_{5}$.

### 3.5. Stability Analysis

Section 3.2, Section 3.3 and Section 3.4 introduced the controllers as parts of the proposed approach. The Lyapunov candidates of each part are designed, and the stability of them are proved separately. To prove the stability of the whole proposed controller, a Lyapunov function that includes all of the Lyapunov candidates of the sub-controllers should be designed. Consider the following Lyapunov candidate:

(38) $$\begin{array}{cc} V & =V_{1}+V_{3}+V_{5}\\ \; & =\frac{1}{2}\left(p_{e}^{2}+e_{\psi ,1}^{2}+s_{1}^{2}+e_{v,1}^{2}+s_{2}^{2}\right) \end{array}$$

where $V_{1}$, $V_{3}$, and $V_{5}$ are all quadratic, and they are added together, so there are no extra parameters in *V*. This means that the Lyapunov candidate *V* has included all of the characteristics of the sub-controller candidates, and its stability can be easily proved.

Applying (10), (24), and (37) into (39) yields

(39) $$\begin{array}{cc} \dot{V} & \leq -\sigma_{1}V_{1}-\sigma_{3}V_{3}-\sigma_{5}V_{5}\\ \; & \leq -\sigma V \end{array}$$

where $\sigma =\min[\sigma_{1},\sigma_{3},\sigma_{5}]$.

Therefore, the conclusion can be drawn that location deviations $p_{e}$ and $e_{\psi ,1}$, $e_{\psi ,2}$, $s_{1}$, $e_{v,1}$, $e_{v,2}$, and $s_{2}$ are bounded and can be eliminated to a small neighborhood around zero.

### 3.6. Optimization Algorithm

#### 3.6.1. Traditional PSO and GWO Algorithm

- In traditional PSO, a set of $n\in Z^{+}$ particles is defined with particles in each iteration, such as a certain *i*th particle in the *k*th iteration with a velocity and a position, separately denoted by $v_{i}\left(k\right)$ and $x_{i}\left(k\right)$. The updating law of the particles is designed as
  (40) $$\begin{array}{cc} \; & v_{i}\left(k+1\right)=wv_{i}\left(k\right)+c_{1}r_{1}\left(k\right)\left[p_{i}\left(k\right)-x_{i}\left(k\right)\right]+c_{2}r_{2}\left(k\right)\left[p_{g}\left(k\right)-x_{i}\left(k\right)\right]\\ \; & x_{i}\left(k+1\right)=x_{i}\left(k\right)+v_{i}\left(k\right) \end{array}$$
  in which $p_{i}$ is the best position in the position history of the *i*th particle, $p_{g}$ is the global best position of all the particles, $c_{1}$ is the self-learning factor, $c_{2}$ is the team-learning factor, and *w* is the inertial weight. Normally, the parameters of *w*, $c_{1}$, and $c_{2}$ are set to constant values, while $r_{1}\in \left(0,1\right)$ and $r_{2}\in \left(0,1\right)$ are random numbers. Although the PSO algorithm is logically simple and fast in finding the optimal solution, it often converges directly when a local optimal solution is found while ignoring the global optimal solution. This is often referred to as the local optimum problem and indicates that there is still room for improvement.
- The optimization algorithm GWO is inspired by the hunting process of wolves. In the traditional GWO algorithm, there are N individuals in D-dimensional space at $X=\left(x^{1},x^{2},\ldots ,x^{D}\right)$, which represents the grey wolf population. Define the solution with the best result as α and the solutions with the second and third best result as β and δ. Then, a candidate would be defined as ω, the whole hunting process is led by the leading wolves, and the candidate ω will follow them. First, they will track and chase their goal, and the action can be described as follows:
  (41) $$\begin{array}{cc} \; & D=\left|C\cdot X_{p}\left(k\right)-X\left(k\right)\right|\\ \; & X\left(k+1\right)=X_{p}\left(k\right)-A\cdot D \end{array}$$
  where *D* is the distance between the prey and grey wolves, $X_{p}$ is the position of the prey, and *X* is the position of the wolves. A=2a·rand(0,1)−a and C=2·rand(0,1). *a* is one of the factors that decrease linearly from two to zero. Then, they will start to hunt, and α, β, and δ will lead the grey wolf population to surround the prey, which can be described as follows:
  (42) $$\begin{array}{cc} \; & D_{\alpha }=\left|C_{1}\cdot X_{\alpha }-X\right|\\ \; & D_{\beta }=\left|C_{2}\cdot X_{\beta }-X\right|\\ \; & D_{\delta }=\left|C_{3}\cdot X_{\delta }-X\right| \end{array}$$
  where $D_{\alpha }$, $D_{\beta }$, and $D_{\delta }$ represent the distance between α, β, and δ and the candidates. $C_{1}$,$C_{2}$, and $C_{3}$ are random vectors, and *X* is the position of the current wolf. Now, we define
  (43) $$\begin{array}{cc} \; & X_{1}=X_{\alpha }-A_{1}\cdot \left(D_{\alpha }\right)\\ \; & X_{2}=X_{\beta }-A_{2}\cdot \left(D_{\beta }\right)\\ \; & X_{3}=X_{\delta }-A_{3}\cdot \left(D_{\delta }\right). \end{array}$$
  Then, the position of the wolf in the next step can be calculated as
  (44) $$X\left(k+1\right)=\frac{X_{1}+X_{2}+X_{3}}{3}$$
  Therefore, the current grey wolf will move around the prey and get close to it. In Equation (41), *A* is a random factor in the range of (−2a,2a); when |A| is greater or equal to one, the grey wolf will keep searching for a better solution, and when |A| is smaller than one, then the grey wolf will be forced to attack the prey. This method can avoid the local optimization problem.

#### 3.6.2. Improvement in PSO Algorithm

- **Improvement in inertial weight**
  In the PSO algorithm, inertial weight is considerably important for reaching an optimal trade-off between local search and global search, determining how the movements of the previous particle will be propagated to the current particle. On one hand, low values will reduce the influences of previous particles, encouraging the current particle to search in different directions and regions. This kind of behavior can be regarded as exploitation. On the other hand, high values will keep the current particle searching in the same direction. Compared with the behaviors of low values, this process can be considered as an exploration. In order to reach an ideal compromise between the search accuracy and the search speed, we propose a three-stage inertial weight update law as (45):
  (45) $$w\left(k\right)=\left\{\begin{array}{c} w_{\mathrm{start}},\;\left(0<k⩽\frac{1}{3}k_{\max}\right)\\ \left(w_{\mathrm{start}}-w_{\mathrm{end}\;}+1\right)\;\mathrm{rand}(1)\;+0.3,\;\left(\frac{1}{3}k_{\max}<k⩽\frac{2}{3}k_{\max}\right)\\ w_{\mathrm{end}},\;\left(\frac{2}{3}k_{\max}<k⩽k_{\max}\right) \end{array}\right.$$
  where $w_{\mathrm{start}}$ is a relatively large value, $w_{\mathrm{end}}$ is a relatively small value, rand(1) is a random value in the range of 0 to 1, *k* is the number of current iteration, and $k_{\max}$ is the maximum iteration number. As shown in (45), the particles are initialized with a relatively high inertial weight value at the early stage $\left(0<k⩽\frac{1}{3}k_{\max}\right)$, ensuring a fast search for the optimal value. Meanwhile, the assigned high initial values prevent the particle weight from becoming too low in the later iterations and falling into the local optimum. During the middle phase $\left(\frac{1}{3}k_{\max}<k⩽\frac{2}{3}k_{\max}\right)$, we adopt a random update approach for the inertial weight to achieve a balanced exploratory and a smooth exploitative search procedure. The core of the weight update function here consists of an assigned interval bounded by $w_{\mathrm{start}}$ and $w_{\mathrm{end}}$ and its multiplier random number *rand*(1) ∈(0,1). The proposed update law can help to avoid premature convergence. In the close-out iterations $\left(\frac{2}{3}k_{\max}<k⩽k_{\max}\right)$, the particle weight values will be reduced to a relatively low value, maintaining a conservative search for the optimal solution. The proposed three-stage inertial weight update law allows for an enhanced optimization performance.
- **Improvement in learning factor**
  $c_{1}$ can be interpreted as the impact of each particle’s previous experiences, while $c_{2}$ is the influence of global learning. If $c_{1}>0,c_{2}=0$, all particles perform the search independently. In the circumstance of a high value $c_{1}$ and a low value $c_{2}$, the particles focus mainly on searching for their best solutions, leading to divergence. In contrast, if $c_{1}=0,c_{2}>0$, the particles will all reach the same solution. Combining a low value $c_{1}$ and a high value $c_{2}$ will result in a sub-optimal solution in the search. Inappropriate learning factor value will lead to poor performance of the PSO algorithm. Therefore, the new learning factor is designed as (46) and (47).
  (46) $$c_{1}\left(k\right)=e^{\left(\frac{P_{i}\left(k\right)-P_{g}\left(k\right)}{\left|P_{g}\left(k\right)+\varepsilon \right|}\right)}$$
  (47) $$c_{2}\left(k\right)=e^{\left(-p_{i}\left(k\right)+p_{g}\left(k\right)\right)}$$
  where $p_{i}\left(k\right)$ is the historical individual optimum in the k-th iteration, $p_{g}\left(k\right)$ is the population optimum in the *k*-th iteration, and ε is a small constant value. We adopt $p_{g}$ as a penalty term in the update function of $c_{1}\left(k\right)$ to control its value within a reasonable interval and vice versa. The exponential form is introduced to increase the sensitivity to the variations in the fitness values.

#### 3.6.3. Improvement in GWO Algorithm

Traditional grey wolf algorithms converge slowly and tend to fall into local optima. In the flow of the algorithm, the algorithm keeps selecting the three best-positioned wolves in the pack as the alpha wolf, and the pack approaches the direction of the alpha wolf. However, the wolves do not have a concept of speed when searching for prey, which means that the wolves are likely to travel too fast as they approach the prey and fail to find better prey as they go. Additionally, grey wolves do not keep track of where they have reached individually during their travels. These problems will tend to lead traditional grey wolf algorithms into local optimal problems. In the PSO algorithm, the particles update their velocity and position based on the individual optimum and the global optimum. Therefore, inspired by the PSO algorithm, in this study, the grey wolves will have memory, that is, they will calculate their own optimal solution during the hunting process and calculate the solution with the best position in the whole population, the grey wolves will travel at a certain range of speed, and the whole process can be described as follows:

(48) $$\begin{array}{cc} \; & v_{i}\left(k\right)=X_{p}\left(k\right)-A\cdot \left|C\cdot X_{p}\left(k\right)-X_{i}\left(k\right)\right|\\ \; & X_{i}\left(k+1\right)=X_{i}\left(k\right)+v_{i}\left(k\right) \end{array}$$

where $v_{i}\left(k\right)$ is the velocity of the ith wolf, $X{\left(k\right)}_{g}$ is the global best solution of all the wolves, and $X{\left(k\right)}_{ip}$ is the personal best solution of the ith wolf. $X_{p}\left(k\right)$ is the leading wolves’ solution, which is a value calculated from the global best solution and the personal best solution of a current wolf, which is described as follows:

(49) $$X_{p}\left(k\right)=\frac{X_{g}\left(k\right)+X_{ip}\left(k\right)}{2}$$

#### 3.6.4. Optimization of Controller

The cost function is designed as

(50) $$J=\int_{0}^{\infty }a\left|e_{\psi ,1}\left(t\right)\right|+b\left|\delta_{d}\left(t\right)\right|+c\left|e_{v,1}\left(t\right)\right|+d\left|F_{d}\left(t\right)\right|dt$$

where a,b,c,d>0. Additionally, the optimization objective is the control parameters including $k_{\psi ,1}$, $k_{\psi ,2}$, $k_{\psi ,3}$, $k_{v,1}$, $k_{v,2}$, $k_{v,3}$, $\kappa_{1}$, $\kappa_{2}$, $\alpha_{1}$, $\beta_{1}$, $\alpha_{2}$, and $\beta_{2}$.

With these strategies, we use the proposed optimization algorithm to optimize the controller. The optimization process will be illustrated by the following pseudo-code (see Algorithms 1 and 2):
**Algorithm 1:** IPSO.
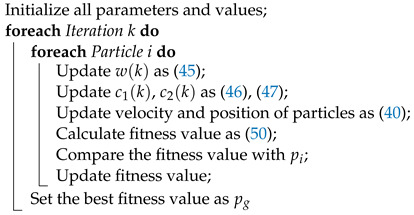

**Algorithm 2:** PGWO.
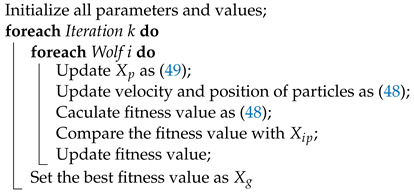


## 4. Simulation Results and Discussion

In this section, leveraging Matlab/Simulink, the performance of the proposed controller is evaluated through three representative examples. The proposed control algorithm was also compared against two different types of controllers, including a PID controller and an SMC controller.

The parameters of this control scheme are listed in Table 1.

### Simulation on a Complex Trajectory

**Figure 3:**
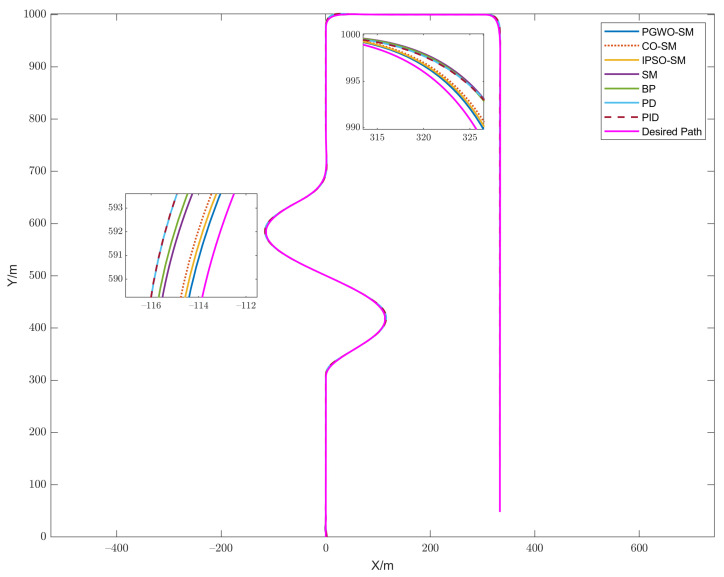
Trajectory tracking comparison.

**Figure 4:**
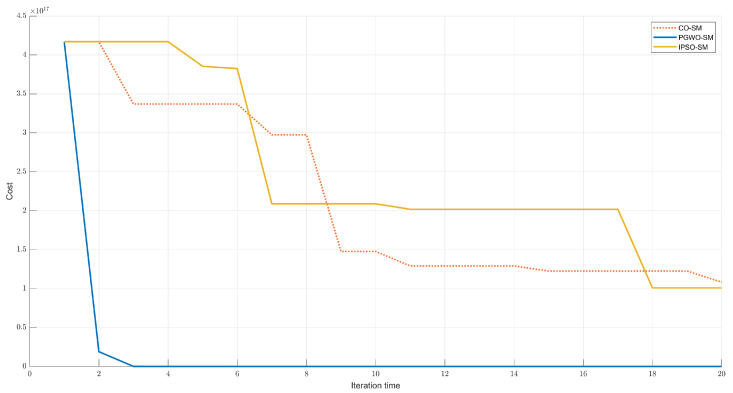
The cost function value.

**Figure 5:**
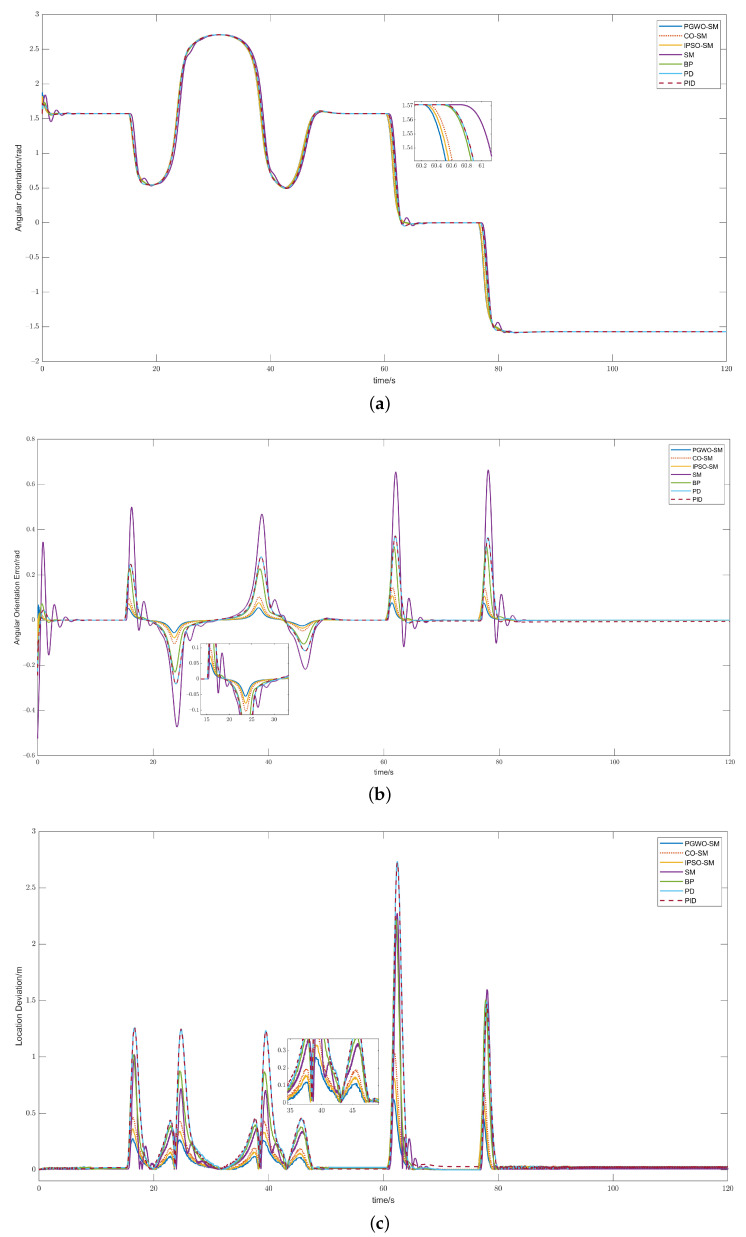
Simulation results of complex trajectory at 20 m/s: (**a**) angular orientation, (**b**) angular orientation error, and (**c**) location deviation.

The test scenario is a complicated trajectory. We set the velocity of the controlled vehicle to 20 m/s. We compared the proposed method with other widely used algorithms such as cuckoo search optimization-based Sliding mode control (CO-SM), sliding mode control (SM), backstepping control (BP), PD control, and PID control. The trajectory tracking result is shown in Figure 3. The cost function value of different evolutionary algorithms is shown in Figure 4. Figure 5a–c demonstrates the angular orientation error and the location deviation.

In Figure 3, it can be seen that the optimized controllers have better performances than the traditional controllers. The PGWO-SM controller has the smoothest and best response. In second and third place are the IPSO-SM and CO-SM controllers. In Figure 4, the cost function value of PGWO-SM is more optimal than IPSO-SM and CO-SM. After three iterations, the PGWO-SM controller has already found the optimal solution, but IPSO-SM and CO-SM can only find their optimal solution at about 20 iterations. Then, it is followed by the unoptimized controllers, in the following order: SM, BP, PID, and PD controllers. Figure 5a,b demonstrate that the optimized controller is more stable than the other controllers in terms of angular orientation. The angular orientation of the PGWO-SM and IPSO-SM controllers are smoother than the SMC and PID controllers, and the angular orientation error of PGWO-SM converges within 1.5 s, which is faster than IPSO-SM and CO-SM, and is significant faster than the other unoptimized controllers; when the vehicle is steered, the error is typically within 0.05 degrees for PGWO-SM and within 0.1 degrees for IPSO-SM and CO-SM. However, the errors for the other traditional controllers are between 0.2 and 0.6 degrees, much greater than the errors for the optimized controller. The local deviation is shown in Figure 5c. When the vehicle is steering at high speed, the PGWO-SM local deviation is usually in the range of 0–0.3 m, with significantly more minor fluctuations in amplitude and duration than the other algorithms. The simulation results show that the optimized controller is significantly more responsive and robust than the conventional SM and PID controllers, while the PGWO-SM controller performs better than the IPSO-SM and CO-SM controllers. To describe the result more clearly, the results are quantified and shown in Table 2. The mean position error and maximal error are calculated here, and this result further proves that our proposed controller is better and can improve the robustness of the system.

In summary, to simulate a complex and hazardous driving scenario, we designed a challenging curved trajectory and controlled the vehicle to travel at high speeds. However, the proposed controller achieves excellent trajectory tracking performance even in such scenarios by minimizing deviations between real and desired trajectories, ensuring rapid convergence even in the presence of large disturbances. Moreover, the controller produces smooth steering angle changes throughout the vehicle’s journey, making it highly suitable for practical vehicle control applications in challenging road conditions.

## 5. Conclusions

We summarized the contributions of the proposed approach as follows: In order to achieve high tracking accuracy in different trajectories, a vector-field-based adaptive sliding mode controller has been presented and described by the bicycle kinematic model. In the proposed method, the vector field guidance law was successfully expanded from simple trajectories to different curves. Additionally, separate control loops for orientation angle and velocity were proposed based on the sliding mode principle. Moreover, two optimization algorithms named IPSO and PGWO were introduced to optimize the sliding mode controller so that the controller was able to output the optimal orientation angle and velocity. We conducted a comparison with the other four algorithms, and the simulation results illustrated that the proposed controller had a faster, smoother, and more precise response compared with the traditional controllers. However, evolutionary algorithms are more demanding in terms of arithmetic power. Furthermore, in this paper, the gain in the controller is searched by the optimization algorithm offline. In the future, more efficient optimization algorithms should be developed, and an online optimization algorithm-based controller for trajectory tracking should be built. Integrated with the research of the longitudinal control approaches, we are going to apply the proposed scheme in a collision avoidance system and eventually apply it to a real car. 

## Figures and Tables

**Table 1:** Main parameters of the controller.

Parameter	Value	Units	Parameter	Value	Units
$k_{\psi ,1,min}$	0	−	$\alpha_{1,min}$	0	−
$k_{\psi ,1,max}$	0.005	−	$\alpha_{1,max}$	1	−
$k_{\psi ,2,min}$	0	−	$\beta_{1,min}$	0	−
$k_{\psi ,2,max}$	10	−	$\beta_{1,max}$	1	−
$k_{\psi ,3,min}$	0	−	$\alpha_{2,min}$	0	−
$k_{\psi ,3,max}$	10	−	$\alpha_{2,max}$	1	−
$k_{v,1,min}$	0	−	$\beta_{2,min}$	0	−
$k_{v,1,max}$	0.005	−	$\beta_{2,max}$	1	−
$k_{v,2,min}$	0	−	$w_{start}$	0.8	−
$k_{v,2,max}$	10	−	$w_{end}$	0.5	−
$k_{v,3,min}$	0	−	$k_{max}$	20	−
$k_{v,3,max}$	10	−	ε	$e^{-50}$	−
*a*	10,000	−	*b*	0.1	−
*c*	0.1	−	*d*	0.001	−

**Table 2:** Comparison of mean value and maximal position error for the simulation results.

Controller	Mean Position Error (m)	Maximal Position Error (m)
PGWO-SM	0.0434	0.6229
CO-SM	0.0640	1.0375
IPSO-SM	0.0529	0.8137
SM	0.0942	2.2759
BP	0.1161	2.2262
PD	0.1507	2.7368
PID	0.1511	2.7215

## Data Availability

The data used to support the findings of this study are available from the corresponding author upon request.
